# Renal Response to L-Arginine in Diabetic Rats. A Possible Link between Nitric Oxide System and Aquaporin-2

**DOI:** 10.1371/journal.pone.0104923

**Published:** 2014-08-11

**Authors:** María C. Ortiz, María F. Albertoni Borghese, Sabrina E. Balonga, Agustina Lavagna, Ana L. Filipuzzi, Rosana Elesgaray, María A. Costa, Mónica P. Majowicz

**Affiliations:** 1 Cátedra de Biología Celular y Molecular, Departamento de Ciencias Biológicas, Facultad de Farmacia y Bioquímica, Universidad de Buenos Aires. Buenos Aires, Argentina; 2 Cátedra de Fisiología, Departamento de Ciencias Biológicas, Facultad de Farmacia y Bioquímica, Universidad de Buenos Aires. Buenos Aires, Argentina; Aarhus University, Denmark

## Abstract

The aim of this study was to evaluate whether L-Arginine (L-Arg) supplementation modifies nitric oxide (NO) system and consequently aquaporin-2 (AQP2) expression in the renal outer medulla of streptozotocin-diabetic rats at an early time point after induction of diabetes. Male Wistar rats were divided in four groups: Control, Diabetic, Diabetic treated with L-Arginine and Control treated with L-Arginine. Nitric oxide synthase (NOS) activity was estimated by [^14^C] L-citrulline production in homogenates of the renal outer medulla and by NADPH-diaphorase staining in renal outer medullary tubules. Western blot was used to detect the expression of AQP2 and NOS types I and III; real time PCR was used to quantify AQP2 mRNA. The expression of both NOS isoforms, NOS I and NOS III, was decreased in the renal outer medulla of diabetic rats and L-Arg failed to prevent these decreases. However, L-Arg improved NO production, NADPH-diaphorase activity in collecting ducts and other tubular structures, and NOS activity in renal homogenates from diabetic rats. AQP2 protein and mRNA were decreased in the renal outer medulla of diabetic rats and L-Arg administration prevented these decreases. These results suggest that the decreased NOS activity in collecting ducts of the renal outer medulla may cause, at least in part, the decreased expression of AQP2 in this model of diabetes and constitute additional evidence supporting a role for NO in contributing to renal water reabsorption through the modulation of AQP2 expression in this pathological condition. However, we cannot discard that another pathway different from NOS also exists that links L-Arg to AQP2 expression.

## Introduction

The imbalance of different vasoactive substances contributes to the development and progression of complications in Diabetes Mellitus (DM). For example, nitric oxide (NO) synthesis is found to be abnormal [1] and several studies have provided evidence involving diabetes as a state of renal NO deficiency [2].

L-arginine (L-Arg) is a semi-essential amino acid and also the main source for the generation of NO via nitric oxide synthase (NOS) [3]. The kidney plays a key role in Arg metabolism and the enzymes that participate in L-Arg synthesis are down-regulated in DM, impairing L-Arg production quite early before any significant structural damage is evident, thus contributing to the resulting NO deficiency [4]. It has been also demonstrated that diabetic animals have increased hepatic Arg degradation resulting in reduced plasma Arg levels, which limit the renal medullary NO levels [5].

On the other hand, serum levels of asymmetric dimethylarginine (ADMA), an endogenous inhibitor of NOS, are increased in both type 1 and type 2 DM, which leads to a decrease in NO production and thus contributes to diabetic complications such as nephropathy and retinopathy [6].

As DM is associated with reduced plasma levels of Arg, L-Arg supplementation may be effective in the improvement of the endothelial and renal function in diabetic patients [7].

The three NOS isoforms are expressed in the kidney; these isoforms are neuronal NOS (nNOS; NOS I); endothelial NOS (eNOS; NOS III) and inducible NOS (iNOS; NOS II), being NOS I and NOS III the main isoforms involved in tubular sodium and water transport regulation [8]. The renal medulla, where the three isoforms are expressed, has a greater ability to generate NO than the renal cortex [9].

Different studies have shown an altered expression of multiple medullar water and sodium transporters in the kidney of DM I rats, being aquaporin-2 (AQP2) one of these transporters [10]. AQP2, a water channel mainly regulated by vasopressin, is localized in the principal cells of the collecting ducts and constitutes the final checkpoint for renal water reabsorption [11]. In addition to vasopressin, several other factors affect AQP2 transcription. Feraille et al. recently described a novel role for NADPH oxidase 4 (NOX4) in regulating AQP2 abundance. They proposed that NOX4-mediated production of reactive oxygen species (ROS) may enhance V_2_R-cAMP-PKA signaling by attenuating phosphodiesterase (PDE) activity [12]. It is interesting to note that in physiological conditions renal tubular NOX4 produces low amounts of ROS that stimulate intracellular signal pathways, but in a rodent model of streptozotocin (STZ)-induced type 1 diabetes, renal expression of NOX4 is increased, and this is associated with ROS-induced renal damage [13].

There are controversial findings about AQP2 expression in diabetes induced by STZ. Some studies have shown an increase in both AQP2 expression and translocation in the renal medulla [14], whereas others have shown either a decrease [15] or no changes [16] in the expression of the protein. These results have all been found after the first two weeks of development of diabetes and there are no studies showing AQP2 expression at an early time point after the induction of diabetes.

There is evidence that NO can modulate AQP2 translocation to the plasma membrane [17]–[19], but there is very scarce information about NO contribution to AQP2 expression. Boone et al., using mpkCCD cells, demonstrated that the activators of the cGMP pathway ANP, L-Arg and 8-Br-cGMP do not induce AQP2 expression in vitro by themselves but they induce AQP2 translocation to the apical membrane. However, when they tested the effect of 1-desamino-8-D-arginine vasopressin (dDAVP) combined with activators of the cGMP pathway on AQP2 expression in the mpkCCD cells, only L-Arg seemed to enhance AQP2 expression. They suggested that the beneficial effect of compounds of the cGMP pathway to relieve nephrogenic diabetes insipidus may be improved when combined with agents that stimulate AQP2 expression [20].

It has been shown that both mRNA and protein of all NOS isoforms are increased in the renal medulla of rats with four days of water deprivation [21], which suggests that the intrarenal NO system is very important in the control of body volume balance. In addition, it has been suggested that endogenous NO produced from NOS I may participate in the regulation of water reabsorption by the collecting duct [22].

On the other hand, Morishita et al. have shown that KO mice lacking all NOS isoforms develop nephrogenic diabetes insipidus with a reduced antidiuretic response to exogenous vasopressin, accompanied by impaired renal cAMP production, a defective membrane expression of AQP2 water channel, and tubuloglomerular lesion [23]. These antecedents prompted us to evaluate whether L-Arg supplementation modifies NOS I and NOS III expression and activity and consequently AQP2 expression in the renal outer medulla of STZ-induced diabetic rats at an early time point. We intended to find new evidence supporting a role for the NO system in AQP2 expression.

Our hypothesis was that AQP2 expression is already decreased in diabetic animals at an early time point after induction of diabetes and that L-Arg supplementation improves NO production, which, in turn, increases AQP2 expression.

## Materials and Methods

### Chemicals

The reagents used in western blot analysis were from Bio Rad (CA, USA). All other chemicals and reagents, unless otherwise indicated, were from Sigma-Aldrich Corporation (St. Louis, MO, USA).

### Animals and treatments

All experimental protocols were performed in accordance with the Guide for the Care and Use of Laboratory Animals (National Institutes of Health, Publication No. 85-23, Revised 1996) and Regulation No. 6344/96 of Argentina’s National Drug, Food and Medical Technology Administration (ANMAT). Experimental procedures were approved by the Ethics Committee of the School of Pharmacy and Biochemistry, University of Buenos Aires.

Male Wistar rats weighing 250–300 g were purchased from the facilities of the School of Pharmacy and Biochemistry of the University of Buenos Aires (Buenos Aires, Argentina) and were housed in a humidity and temperature-controlled environment with an automatic 12-hour light-dark cycle. They were fed standard rat chow and provided tap water ad libitum up to the day of the experiments. The animals were divided in four groups: Control, Diabetic, Diabetic treated with L-Arg, and Control treated with L-Arg. Diabetes was induced by a single intraperitoneal injection of STZ in a dose of 70 mg/kg of body weight diluted in citrate buffer [24]. Controls were injected with citrate buffer alone. L-Arg was administered to the diabetic treated and control treated groups in the drinking water in a dose of 622 mg/kg/day [25], simultaneously with the induction of diabetes. The concentration of L-Arg in the drinking water was adjusted considering the drinking water intake from the previous day for each animal to eliminate changes due to different intakes.

Four days after the induction of diabetes, the animals were anesthetized with ether and blood samples were immediately obtained by cardiac puncture until exsanguinations and all efforts were made to minimize suffering.

Four days were chosen because at this early time, the animals already manifest hyperglycemia and polyuria and it is a time long enough to allow changes in protein abundances but too short to lead to large effects resulting from diabetic nephropathy. Besides, there are no results regarding AQP2 expression at this early time point after the onset of diabetes. On the other hand, long term arginine supplementation is ineffective in improving NOSIII activity and even it may be detrimental [26].

Glycemia was measured in blood samples by the glucose oxidase method (Betachek, National Diagnostic Products, Sidney, Australia). The diabetic condition was characterized by a clear hyperglycemic state, loss of body weight and increased diuresis four days after STZ injection. STZ-treated animals with glucose plasma levels below 11 mmol/l were not included in the experimental protocol.

### Determinations in the 24-hour metabolic cage studies

Twenty four-hour urine samples were collected using metabolic cages. Animals were allowed to acclimatize to metabolic cages for two days and then fasted for 24 h before the collection of urine to avoid differences in plasma glucose concentration and NOx excretion due to different food intake. Urine samples (24 h) were analyzed for total protein by a turbidimetric method using trichloroacetic acid as previously described [27]. Urine analysis for glucose and ketones was carried out using reactive strips (Keto-Diastix Bayer Diagnostics S.A., Argentina). Urine osmolality was measured with an osmometer (OSMETTE, Precision Systems Inc., MA, USA) and expressed as osmolar excretion rate. Urine volume was measured gravimetrically. Kinetic determinations of serum and urinary creatinine concentration were evaluated using a kit provided by Wiener (Wiener Lab., Rosario, Argentina).

### Tissue processing for Western blot analysis

Immediately after the animals were sacrificed, their kidneys were isolated and the outer medulla was dissected and homogenized at 3.000 rpm in an appropriate buffer (250 mmol/l sucrose, 1 mmol/l EDTA, 0.1 mmol/l PMSF and 10 mmol/l Tris-ClH), pH 7.6. Large tissue debris and nuclear fragments were removed by a low-speed spin (1000 g, 10 min, 4°C). Protein concentration was measured using BCA™ Protein Assay Kit (Pierce, Rockford, IL, USA). Absorbances for protein concentration measurements were read using an RT-2100C microplate reader (Rayto, China) at 560 nm.

### Western blots of AQP2 and NOS isoforms

Western blot analysis was used to identify AQP2, NOS I and NOS III. Blots were incubated overnight at 4°C with the AQP2 antibody (rabbit anti-rat AQP2; Santa Cruz Biotechnology, Inc., CA, USA) diluted in blocking solution (1∶500), or with NOS I or NOS III antibodies (rabbit anti-rat; diluted 1∶1000; BD Transduction Laboratories, NJ, USA). Beta-tubulin was used as loading control (rabbit anti-rat beta-tubulin; Abcam Inc., Cambridge, MA, USA). The membranes were then incubated with a biotinylated donkey anti-rabbit IgG (1∶2.500) (Jackson ImmunoResearch, Baltimore Pike, Pa., USA). Blots were stained using Vectastain ABC kit and DAB substrate kit for peroxidase (both from Vector Laboratories Inc. Burlingame, CA, USA). The AQP2 antibody recognizes a 28-kDa band corresponding to unglycosylated AQP2 and bands between 35–40 kDa representing glycosylated forms of the protein. The NOS I antibody recognizes a 155-kDa band and the NOS III antibody detects a 140-kDa band.

The relative protein levels were determined by analyzing the bands with Gel Pro Analyzer 3.1 for Windows and protein expression was calculated as the ratio of AQP2, NOS I or NOS III to beta-tubulin.

### Real-time PCR for AQP2

Total RNA was isolated using the SV total RNA Isolation System (Promega, Madison, WI, USA). Total RNA was reverse transcribed to cDNA using a high capacity reverse transcription kit (A&B, CA, USA). For real-time detection of AQP2 transcripts and the reference gene (GAPDH), MezclaReal (Real Time PCR commercial mixture from Biodynamics, Argentina) and specific primers [28] were used. The normalized gene expression method (2^–ΔΔCT^) for the relative quantification of gene expression was used. The difference in the cycle threshold (CT) for AQP2 and GAPDH for the control untreated rats was substracted from the difference in the CT for AQP2 and GAPDH for each of the experimental groups. The following formula was applied: ΔΔCT = (*CTAQP*2−*CTGAPDH*)*experimental*−(*CT AQP*2−*CT GAPDH*)*control untreated rats* [29].

The real-time PCR started at 94°C for 2 min and was followed by 35 thermal cycles at 94°C for 15 s, 58°C for 35 s and 72°C for 30 s.

### Histochemistry

Tissues were processed using the NADPH-diaphorase (NADPH-d) histochemical method according to Rothe et al. [30]. NADPH-d staining is widely used to detect NOS-containing cells in neural and non-neural tissues. With the appropriate fixation procedure, this method can detect cells containing any of the NOS isoforms [31]. The NADPH-d reaction can be used to monitor NOS activity at a cellular level of resolution [32]. The kidneys were fixed with 4% paraformaldehyde in 0.1 M phosphate buffer pH 7.4, then the tissues were cryoprotected with sucrose, frozen, sectioned at 14 µm on a criostate and mounted on gelatin-coated glass slides. Briefly, sections were simultaneously incubated for 1 h at 37°C in the same reaction mixture containing 0.1% β-NADPH and 0.02% nitrobluetetrazolium diluted in 0.1 M phosphate buffer with 0.3% Triton X-100. Then, sections were mounted in PBS/glycerol (1∶3). Observation, optical density (OD) measurement and photography were made with a Nikon Alphaphot-2 YS2 coupled to a SONY camera Model N°SSC-DCSOA. NADPH-d-stained cells from the different groups were measured by a computer image-analysis program (Scion Image). Each set of OD measurements (control and experimental groups) was performed blindly and under similar conditions of light, gain, offset and magnification. After automatic normalization of gray-scale, the same interactive delineation and contrast enhancement of all images was performed. To obtain each value, the program calculates the mean of different OD values obtained in the same renal tubule. This process was done in different renal tubules of the same section and different sections of the same kidney.

### NO metabolites measurement

The concentration of NO metabolites (nitrites and nitrates, NOx) in urine samples was determined according to the procedure described by Verdon et al. [33].

### Nitric oxide synthase activity

Total NOS activity in the renal outer medulla was determined on the basis of the rate of L-[^14^C] citrulline formation from [^14^C] L-Arg, as described previously [34].

Specific NOS activity was assessed in the presence of 10^−4^ M L-nitro-arginine-methyl-ester (L-NAME). NO production (measured as pmol of [^14^C] L-citrulline) in each tube was normalized to the weight of the tissue slices incubated with the substrate for equal periods of time and expressed as pmol/g wet weight/min. Total NOS activity was defined as the [^14^C] L-Arg-to-[^14^C] L-citrulline conversion that was inhibited by the non-selective NOS inhibitor L-NAME (10^−4^ mol/L).

### Statistics

Results are expressed as the mean ± SEM. Two-way ANOVA was used to analyze the data, where one factor was the diabetes (control or diabetic rats) and the other the treatment with L-Arg (treated or untreated rats). The main effect of each factor was tested as well as the interaction within both factors. Bonferroni’s post-test was used for multiple comparisons when interaction was statistically significant. When the interaction was found to be statistically significant, the main effect of each factor was not informed (as each factor is influenced by the other) and simple main effects were informed separately (e.g. the effect of L-Arg on control rats and the effect of L-Arg on diabetic rats, separately). The analysis was performed using Graph Pad Prism version 5.0 for Windows, Graph Pad Software (San Diego, CA, USA). The null hypothesis was rejected when p<0.05.

## Results

### Effects of L-Arg administration on some general and renal parameters

The results of L-Arg administration on some general and renal parameters are shown in Table 1.

**Table 1:** Summary of some general and renal parameters in control and diabetic rats.

	C	D	C+A	D+A
Initial body weight (g)	307.3±11.1	320.6±21.7	311.2±12.0	327.2±20.5
Final body Weight (g)	355.1±17.2	270.1±19.4***	347.9±22.5	275.6±16.1^###^
Kidney weight/body weight (mg/g)	3.7±0.2	5.4±0.4**	4.2±0.4	5.1±0.5^##^
Blood glucose (mmol/l)	5.6±0.5	16.1±1.7***	5.0±0.4	15.7±1.2^###^
Urinary volume (ml/24 h)	10.3±1.1	15.9±1.4*	9.0±1.2	12.9±1.4^#^
Osmolar excretion rate (mOsm/24 h)	8.5±1.1	18.2±2.5**	9.5±1.6	16.3±3.2^##^
Urinary glucose (mmol/l)	Undetectable (0.00±0.00)	33.3±9.4***	Undetectable (0.00±0.00)	30.5±9.1^###^
Urinary ketones (mmol/l)	Undetectable (0.00±0.00)	0.78±0.19***	Undetectable (0.00±0.00)	0.29±0.12^###^
Urinary proteins (mg/24 h)	6.4±0.4	6.9±1.6	6.7±0.3	4.7±0.5
Creatinine clearance (ml/min)	1.09±0.15	1.33±0.12	0.93±0.11	1.12±0.14
NOx (nmol/mg creatinine)	10.6±1.3	5.0±0.6*	11.8±0.7	13.9±2.4^&&&^

C: control rats; D: diabetic untreated rats; D+A: diabetic rats treated with L-Arg; C+A: control rats treated with L-Arg. Results are expressed as the mean ± SEM (n = 6).

Two-way ANOVA showed a statistically significant interaction (p<0.05) between the effects of Diabetes and L-Arg treatment on NOx urinary excretion. There was no interaction between the effects of Diabetes and L-Arg treatment on the other parameters. Diabetes had a significant overall effect on Final body weight (p<0.001), Kidney weight/body weight (p<0.001), Blood glucose (p<0.001), Urinary volume (p<0.05), Osmolar excretion rate (p<0.01), Urinary glucose (p<0.001) and Urinary ketones (p<0.001). L-Arg had no significant effect on any of the parameters tested. *p<0.05 vs. C; **p<0.01 vs. C; ***p<0.001 vs. C; ^#^p<0.05 vs. C+A; ^##^p<0.01 vs. C+A ^###^p<0.001 vs. C+A; ^&&&^p<0.001 vs. D.

The body weight of diabetic rats (untreated and treated with L-Arg) decreased when compared with control groups (untreated and treated with L-Arg, p<0.001). Although the kidney weights of the different groups showed no changes (data not shown), the kidney weight/body weight ratio of the diabetic animals increased due to the decrease in their body weights (p<0.01). These results provide evidence that L-Arg administration did not prevent the loss of weight in diabetic animals.

On day four, blood glucose was increased to the same extent in both diabetic groups. Urinary glucose (mmol/l) was undetectable in control groups, whereas it was 33.3±9.4 in diabetic untreated rats and 30.5±9.1 in diabetic rats treated with L-Arg. These results indicate that L-Arg administration did not correct the levels of blood and urinary glucose in diabetic rats, although it partially corrected the value of urinary ketones.

Urinary volume (ml/24 h) and Osmolar excretion rate increased in diabetic rats and were partially corrected by L-Arg administration, although the values obtained in diabetic rats treated with L-Arg were still higher than those of control and control treated with L-Arg rats.

Urinary protein excretion and creatinine clearance showed no statistically significant changes in any of the groups. L-Arg administration to control rats did not modify any of the parameters tested.

### L-Arg improves urinary NO metabolites production and NADPH-d activity in some structures of the renal outer medulla in diabetic rats

Table 1 shows the urinary excretion of NO metabolites, which traditionally was considered to reflect systemic NO production [35]. However, recently, Hyndman et al. showed that urinary excretion of NO metabolites should be considered a measure of collecting duct-derived NO production [36]. The excretion was normalized to urinary creatinine to minimize possible confounding effects of urinary dilution. The results showed an interaction between the effects of diabetes and L-Arg administration (p<0.05). NOx (nmol/mg creatinine) significantly decreased in the diabetic untreated group compared to the control untreated group. L-Arg administration produced an increase in NOx in diabetic rats but not in control rats.

Table 2 and figures S1 and S2 show the effect of L-Arg on NADPH-d activity in the renal outer medulla of control and diabetic rats. NADPH-d activity (expressed as arbitrary units of optical density) was decreased in the collecting ducts of the diabetic rats present in the outer stripe of the outer medulla and in the thick ascending limb of Henle’s loop present in the inner stripe of the outer medulla, whereas in the collecting ducts of the inner stripe of the outer medulla NADPH-d activity showed a trend (p<0.1) to decrease. L-Arg administration to diabetic rats improved NADPH-d activity in proximal tubules from the outer stripe, in the thick ascending limb of Henle’s loop from the inner stripe and in the collecting ducts from the inner and outer stripe of the outer medulla. On the other hand, L-Arg administration to control rats enhanced NADPH-d staining only in the collecting ducts from both inner and outer stripe.

**Table 2:** Effect of L-arginine on NADPH-diaphorase activity in the renal outer medulla of control and diabetic rats.

	C	D	C+A	D+A
**PT (OS)**	0.235±0.006 (n = 32)	0.241±0.007 (n = 40)	0.244±0.004 (n = 45)	0.298±0.009^###&&&^ (n = 25)
**CD (OS)**	0.171±0.003 (n = 24)	0.142±0.008** (n = 15)	0.209±0.005*** (n = 24)	0.276±0.007^###&&&^ (n = 31)
**CD (IS)**	0.157±0.007 (n = 16)	0.147±0.004 (n = 22)	0.187±0.003*** (n = 19)	0.158±0.004^###&^ (n = 16)
**THAL (IS)**	0.255±0.004 (n = 30)	0.210±0.002*** (n = 32)	0.258±0.003 (n = 32)	0.221±0.002^###&^ (n = 26)

C: control rats; D: diabetic untreated rats; D+A: diabetic rats treated with L-Arg; C+A: control rats treated with L-Arg. PT: proximal tubule; CD: collecting duct; THAL: thick ascending limb of Henle; OS: outer stripe; IS: inner stripe. NADPH-d staining values are expressed as optical density. Results are expressed as the mean ± SEM; “n” represents the total number of renal tubules analyzed. At least four animals of each group were used. Two-way ANOVA showed a statistically significant interaction between the effects of Diabetes and L-Arg treatment on NADPH-diaphorase activity in PT (p<0.001), CD (OS) (p<0.001), CD (IS) (p<0.05) and THAL (p<0.05). Bonferroni’s post-tests are showed in the table: **p<0.01 vs. C; ***p<0.001 vs. C; ^###^p<0.001 vs. C+A; ^&^p<0.05 vs. D; ^&&&^p<0.001 vs. D.

### L-Arg improves NO synthase activity in the renal outer medulla of diabetic and control rats

**Figure 1:**
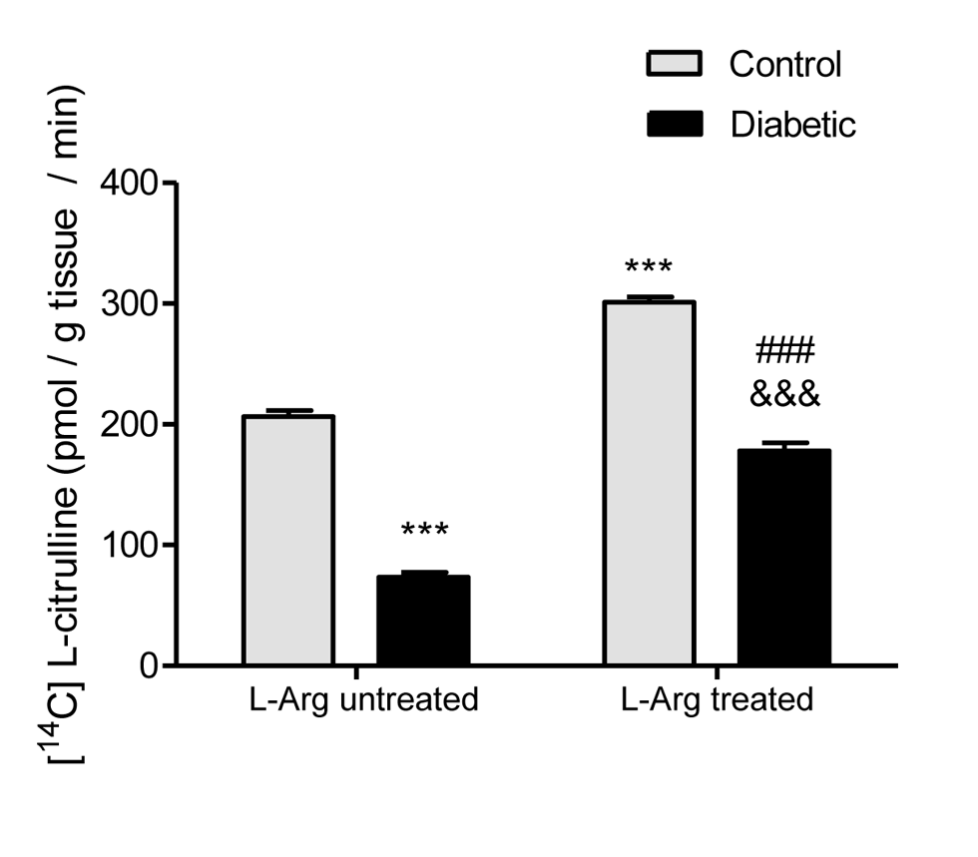
NOS activity measured as pmol [^14^C] L-citrulline/g tissue/min. Two-way ANOVA showed no statistically significant interaction between the effects of Diabetes and L-Arg treatment on NOS activity. The effects of Diabetes and L-Arg were considered extremely significant (p<0.001). ***p<0.001 vs. control untreated rats; ^###^p<0.001 vs. control rats treated with L-Arg; ^&&&^p<0.001 vs. diabetic untreated rats. Data are mean ± SEM (n = 8).

Figure 1 shows that NOS activity expressed as pmol [^14^C] L-citrulline/g tissue/min dramatically decreased in the renal outer medulla of the diabetic rats. On the other hand, L-Arg administration to the animals prevented the decrease in NOS activity in diabetic rats, and enhanced NOS activity in control rats (Fig. 1).

### L-Arg does not prevent the decreased expression of NOS I and NOS III in the renal outer medulla of diabetic rats

**Figure 2:**
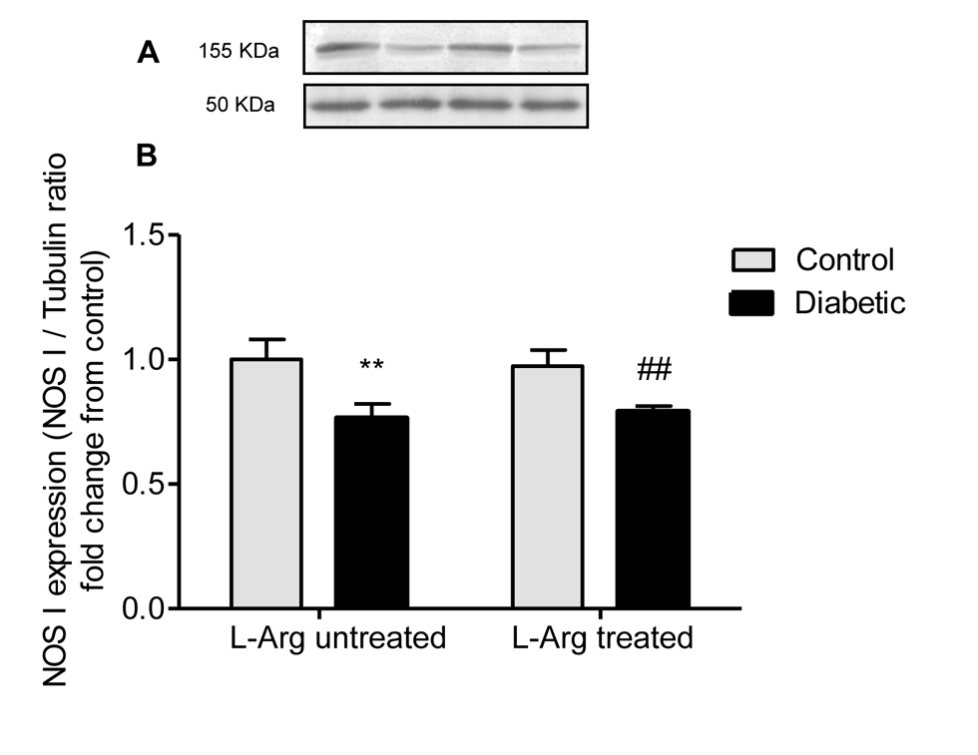
NOS I expression in homogenates of the renal outer medulla. **A.** Representative western blot analysis of NOS I (155 kDa band) and tubulin (50 kDa band) in the renal outer medulla; **B.** NOS I expression indicated as NOS I/tubulin ratio fold change from control untreated rats. Two-way ANOVA showed no statistically significant interaction between the effects of Diabetes and L-Arg treatment on NOS I expression. The effect of Diabetes was considered very significant (p<0.01), the effect of L-Arg was not significant. **p<0.01 vs. control untreated rats, ##p<0.01 vs. control rats treated with L-Arg. Data are mean ± SEM (n = 6).

**Figure 3:**
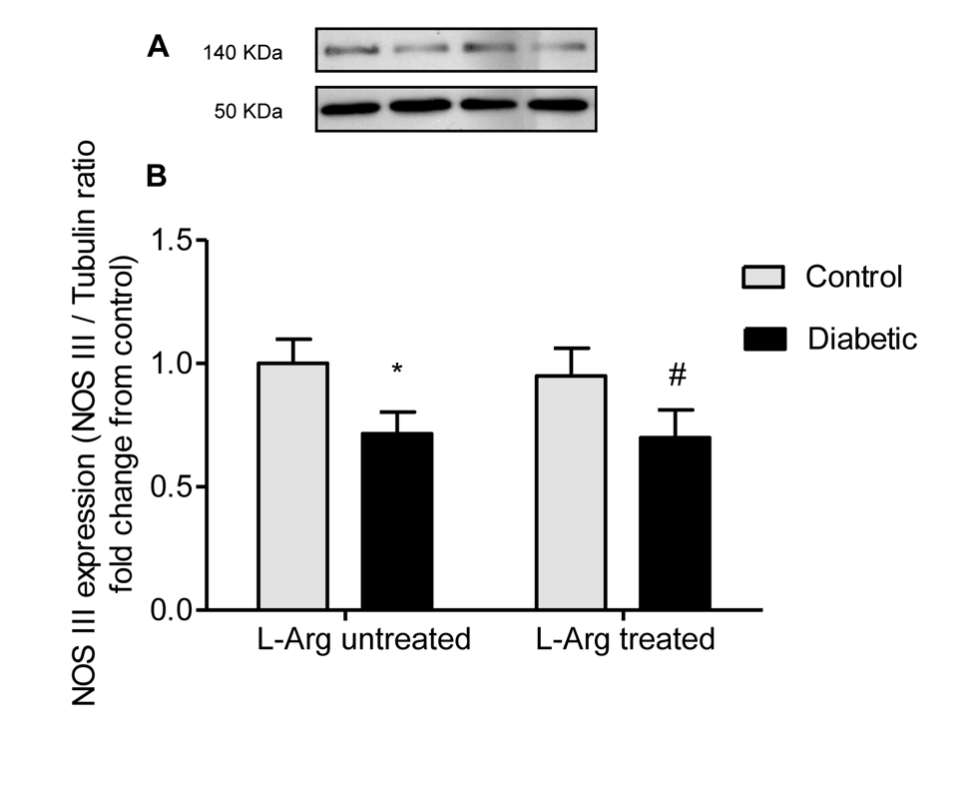
NOS III expression in homogenates of the renal outer medulla. **A.** Representative western blot analysis of NOS III (140 kDa band) and tubulin (50 kDa band) in the renal outer medulla; **B.** NOS III expression indicated as NOS III/tubulin ratio fold change from control untreated rats. Two-way ANOVA showed no statistically significant interaction between the effects of Diabetes and L-Arg treatment on NOS III expression. The effect of Diabetes was considered significant (p<0.05), the effect of L-Arg was not significant. *p<0.05 vs. control untreated rats, #p<0.05 vs. control rats treated with L-Arg. Data are mean ± SEM (n = 6).

Figure 2 and 3 show that the expression of both NOS I and NOS III was decreased in the renal outer medulla of diabetic untreated rats. This result may explain, at least in part, the decreased NADPH-d activity in most renal tubules and the decreased NOS activity measured as [^14^C] L-citrulline production in the diabetic rats. On the other hand, L-Arg administration to diabetic rats did not prevent the decreased expression of NOS I and NOS III (Fig. 2 and 3).

### L-Arg treatment prevents the decrease in AQP2 protein expression and mRNA in the renal outer medulla of diabetic rats

**Figure 4:**
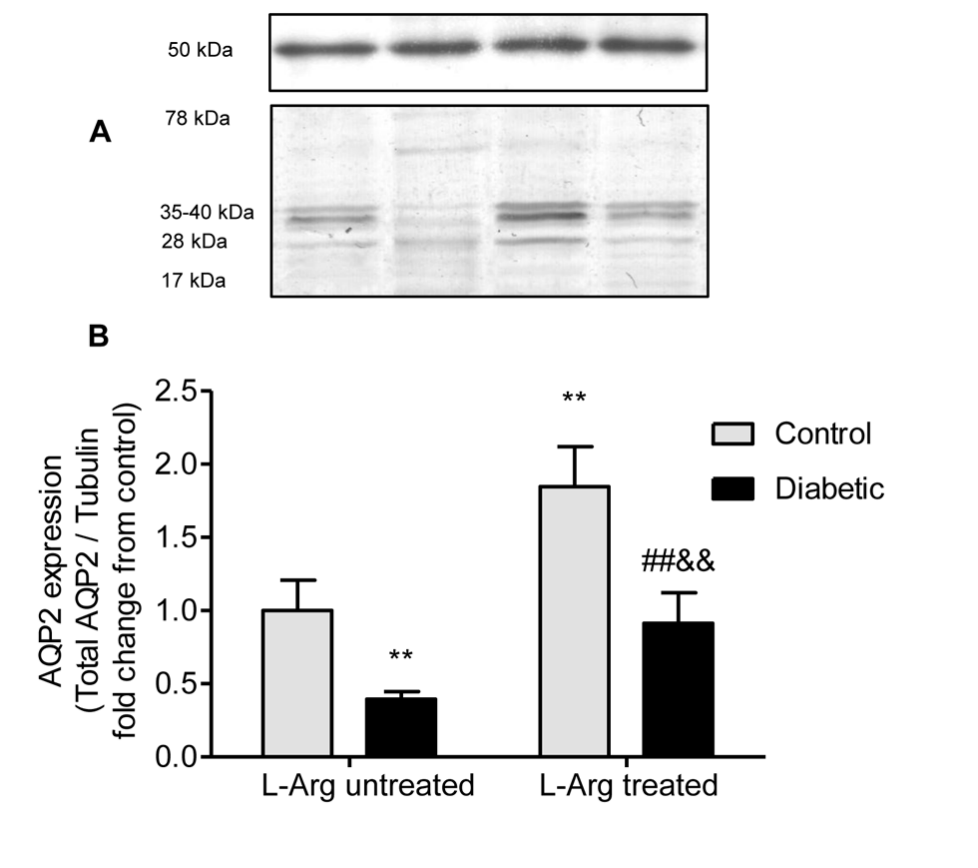
AQP2 expression in homogenates of the renal outer medulla. **A.** epresentative western blot analysis of AQP2 (28 and 35–40 kDa bands; unglycosylated and glycosylated forms respectively) and tubulin (50 kDa band) in the renal outer medulla. **B.** AQP2 expression indicated as the ratio AQP2/tubulin fold change from control untreated rats. Two-way ANOVA showed no statistically significant interaction between the effects of Diabetes and L-Arg treatment on the expression of AQP2. The effects of Diabetes and L-Arg on AQP2 expression were considered very significant (p<0.01). **p<0.01 vs. control untreated rats; ##p<0.01 vs. control rats treated with L-Arg, &&p<0.01 vs. diabetic untreated rats. Data are mean ± SEM (n = 6).

**Figure 5:**
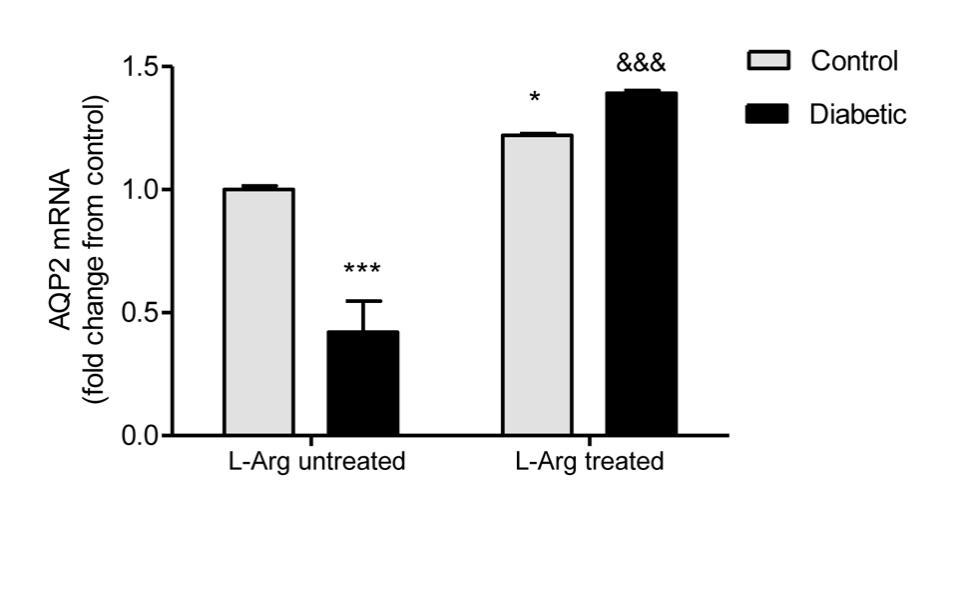
AQP2 mRNA levels in the renal outer medulla. AQP2 mRNA levels are expressed as relative values from control untreated rats. The following formula was applied: ΔΔCT = (*CTAQP*2−*CTGAPDH*) *experimental*−(*CT AQP*2−*CT GAPDH*)*control untreated rats*. Two-way ANOVA showed a statistically significant (p<0.001) interaction between the effects of Diabetes and L-Arg treatment on AQP2 mRNA expression. Bonferroni’s post- tests: *p<0.05 vs. control untreated rats; ***p<0.001 vs. control untreated rats; &&&p<0.001 vs. diabetic untreated rats. Data are mean ± SEM (n = 6).

Figures 4 and 5 show that both AQP2 protein (arbitrary units of total AQP2/tubulin ratio) and mRNA expression were decreased in the renal outer medulla of diabetic untreated rats.

L-Arg administration to the diabetic rats prevented both the decrease in AQP2 mRNA and protein expression, showing values similar to those of the control group for the protein and even greater for the mRNA. It is interesting to note that L-Arg administration increased AQP2 expression to the same extent in control and diabetic rats. Meanwhile, the increase in AQP2 mRNA due to L-Arg administration is higher in diabetic rats than in control rats (Figs. 4 and 5).

The results of AQP2 western blot were corroborated by immunohistochemistry (Figs. S3 and S4), where we showed that AQP2 expression decreased in diabetic rats and that L-Arg treatment increased AQP2 expression in both control and diabetic rats. Additionally, in Figure S4 it can be observed that L-Arg stimulated AQP2 translocation to apical membrane of the principal cells of the collecting ducts, being this effect enhanced in diabetic rats.

## Discussion

In diabetes, abnormalities in NO production impact on renal structure and function [37]. Our study shows that NO production, NOS I and NOS III expression, NOS activity and AQP2 expression are decreased in the renal outer medulla of rats at an early stage of STZ-induced diabetes.

Diabetes is associated with vascular oxidative stress, the activation of NADPH oxidase, and the uncoupling of NOS III [38]. However, results regarding the levels of NOS expression in the kidney of diabetic rats are variable. Some authors have reported an increased expression of the different NOS isoforms [9], whereas others have found a decreased expression [39]. In the present work, we found that the expression of both NOS I and NOS III was decreased in the renal outer medulla of the diabetic rats and that L-Arg administration did not prevent this decrease. Data regarding the impact of L-Arg on the regulation of the expression of genes and proteins for all NOS isoforms are scarce. Rusai et al. have demonstrated that L-Arg supplementation for seven days in rats with a model of renal ischemia increased the mRNA expression of all three NOS isoforms, but increased only NOS II protein levels [40].

On the other hand, we also found an important decrease in NOS activity in the renal outer medulla of the diabetic rats. L-Arg administration prevented the decrease in NOS activity in diabetic animals and increased the activity in control animals.

It is known that NOS activity is regulated by both post-transcriptional and post-translational events and that they may be deranged in pathophysiological states like diabetes [41]. For example, it has been shown that hyperglycemia inhibits NOS III activity by post-translational modification in the Akt site [42]. Bearing in mind that L-Arg did not affect plasma glucose levels, it is unlikely that L-Arg administration prevented the glycosylation of NOS isoforms. On the other hand, hyperglycemia has been associated with increased formation of ROS [43] and it has been reported that L-Arg enhances enzymatic antioxidants in diabetic rats in both the liver and the kidney [44]. So, it may be that L-Arg supplementation enhances NOS activity decreasing ROS formation. This may explain the increase in NOS activity in the diabetic rats, but not in the control rats, which led us to think that L-Arg is more probably acting as an allosteric activator of the enzyme in both control and diabetic rats [45].

Regarding NOx excretion, we showed that there was a significant decrease in the diabetic untreated rats and that the decrease was prevented by L-Arg supplementation. This result correlates with that obtained in NOS activity. However, L-Arg supplementation to control rats only showed a tendency to increase NOx, but this increase was not significant. This may be explained by the fact that NOS activity was measured in homogenates of the renal outer medulla, meanwhile NOx excretion may be reflecting NO systemic production and not only NO renal production.

The early stages of diabetic nephropathy are associated with abnormalities in glomerular hemodynamic as well as with alterations in the renal tubular function. It is known that NO modulates sodium transport along the different segments of the nephron [46], but there is less evidence about the effect of the NO system on water transport.

Our results show that in early diabetes, both AQP2 protein and mRNA are decreased and that L-Arg administration prevented these decreases in the renal outer medulla of diabetic rats.

This is in accordance with a previous work of our laboratory, where we showed that AQP2 is down regulated in the renal medulla of rats made hypertensive by chronic inhibition of NOS [27]. Besides, we have recently demonstrated a synergistic effect of NO and NFATc (nuclear factor of activated T cells) promoting an increase in AQP2 mRNA and protein in mouse papilla and activation of the AQP2 promoter in kidney-derived cells [47].

In opposition to us, a recent work showed an up regulation of glycosylated AQP2 in STZ-diabetic rats and found that the treatment of the animals with L-NAME suppressed the compensatory increase in AQP2 expression [48]. However, that study was performed three weeks after the induction of diabetes and the authors attributed the effect of L-NAME to the inhibition of vasopressin release.

We showed that the increase in the expression of AQP2 mRNA in the diabetic treated group is higher than in the control treated group. However, the higher increase in the diabetic treated group is not reflected at protein level. Numerous observations suggest that AQP2 protein abundance is directly modulated via altered expression of AQP2 mRNA. However, uncoupled expression between AQP2 mRNA and protein has also been described under certain conditions [49]. One explanation of the disparity between AQP2 protein and mRNA expression could be the regulated sequestration of mRNAs that lowers the amount of mRNA available for translation [12], [50]. These observations suggest that AQP2 protein expression may be adjusted not only at the mRNA level but also at a post-transcriptional level.

The present results suggest that the decreased expression and activity of NOS in collecting ducts could be, at least in part, the cause of the AQP2 decreased expression in this model of diabetes induced by STZ. However, NO is known to be diuretic and natriuretic via soluble guanylate cyclase/protein kinase G activation at the proximal tubule level [46]. A possible explanation for these apparently contradictory findings is that at the level of the collecting duct, where the fine tuning of urine composition occurs, an increase in NO may enhance AQP2 in order to avoid an excessive loss of water. In certain pathological states which trigger the development of polyuria, such as diabetes, decreased NO levels would lead to a reduction in the expression of AQP2 in order to avoid an excessive loss of water.

Our results suggest that the decreased NOS activity in the collecting ducts of the renal outer medulla may cause, at least in part, the decreased expression of AQP2 at an early time point in this model of diabetes and constitute additional evidence supporting a role for NO in the contribution to renal water reabsorption through the modulation of AQP2 expression in this pathological condition.

## Supporting Information

Figure S1 (TIF)

Figure S2 (TIF)

Figure S3 (TIF)

Figure S4 (TIF)
