# Supplementary figures and images for: Renal Response to L-Arginine in Diabetic Rats. A Possible Link between Nitric Oxide System and Aquaporin-2
Source: PLoS One. 2014 Aug 11;9(8):e104923. doi: 10.1371/journal.pone.0104923 (PMC4128736; doi:10.1371/journal.pone.0104923)

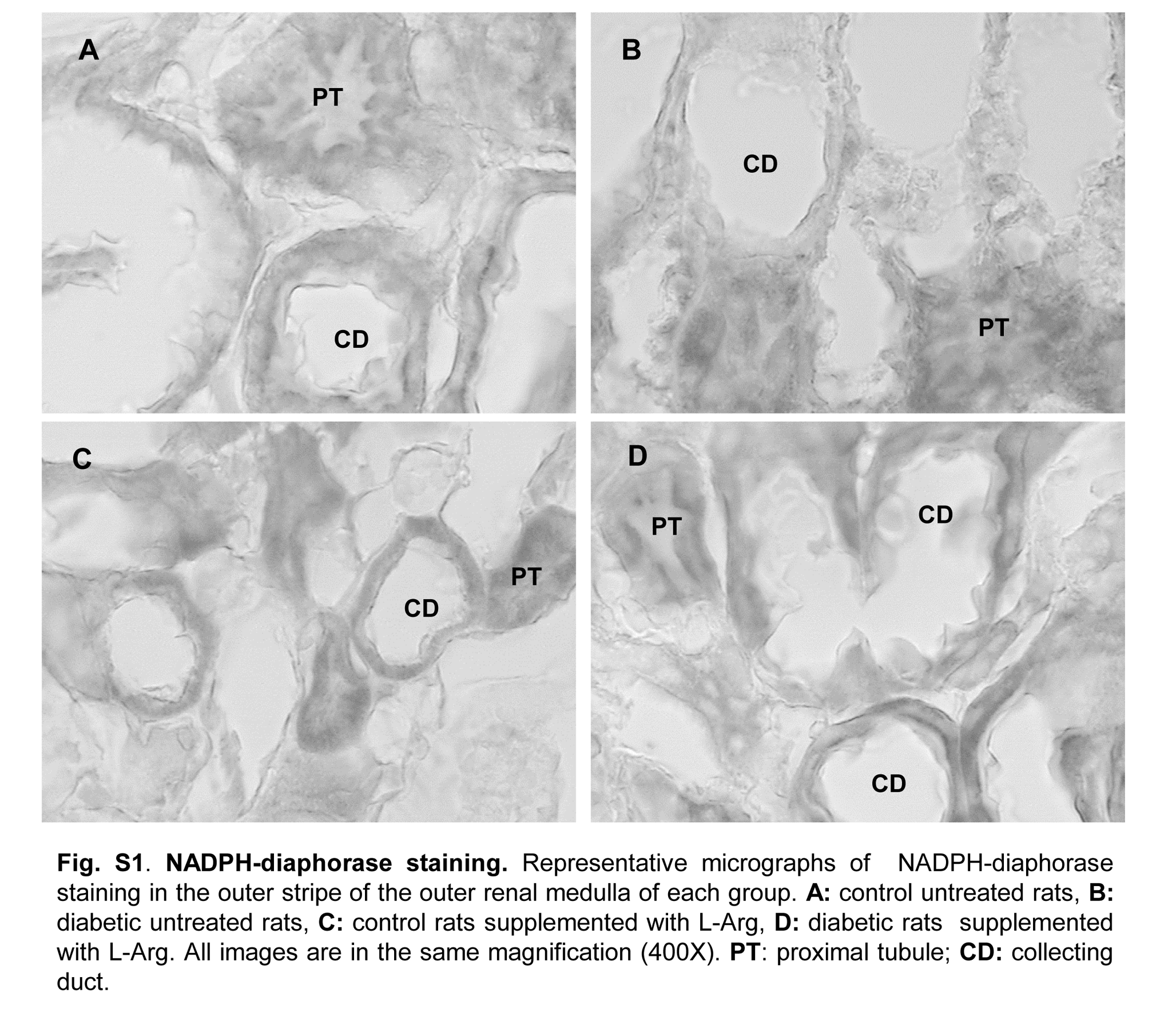

Supplement: Figure S1 — (TIF) [file pone.0104923.s001.tif]

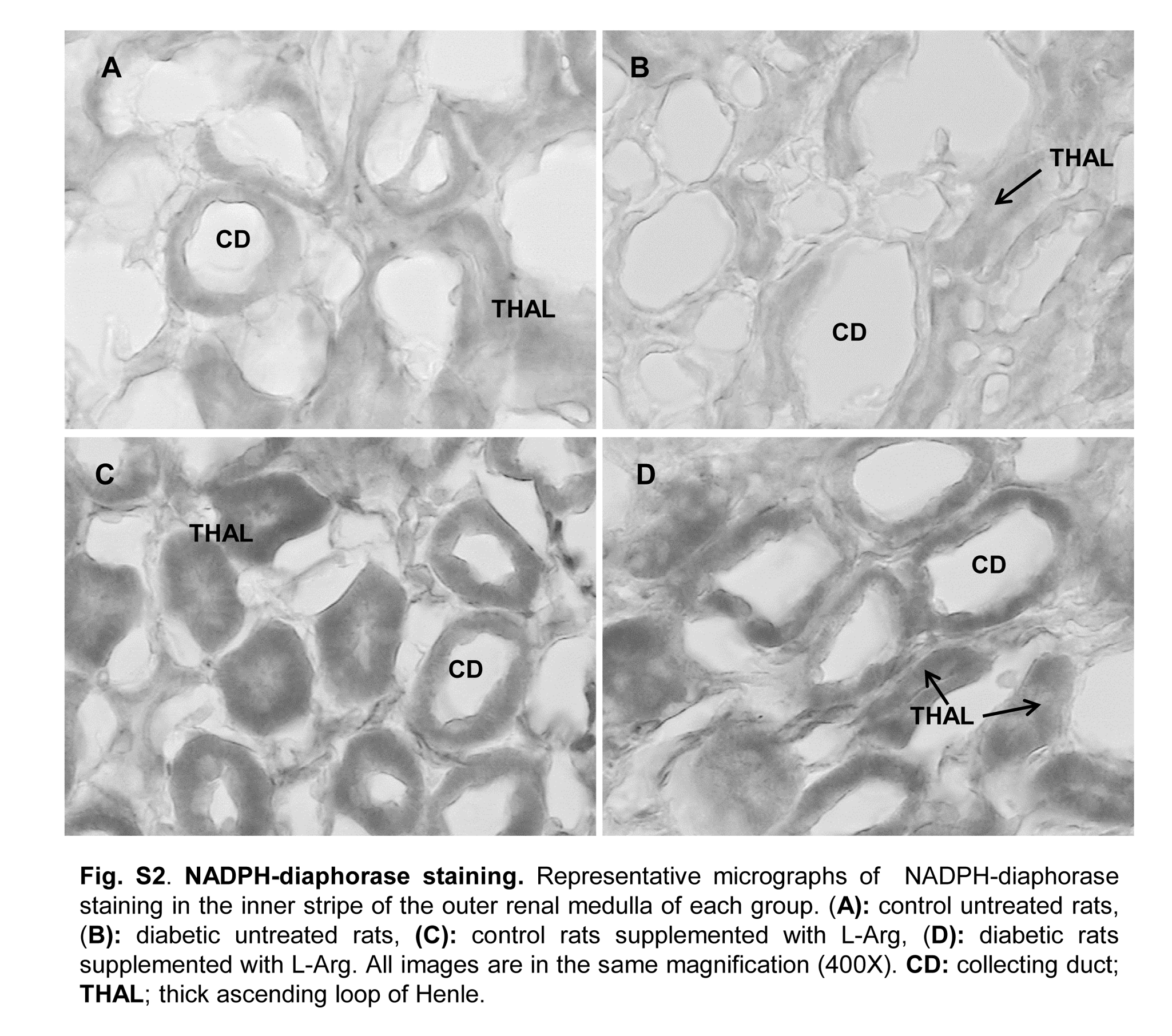

Supplement: Figure S2 — (TIF) [file pone.0104923.s002.tif]

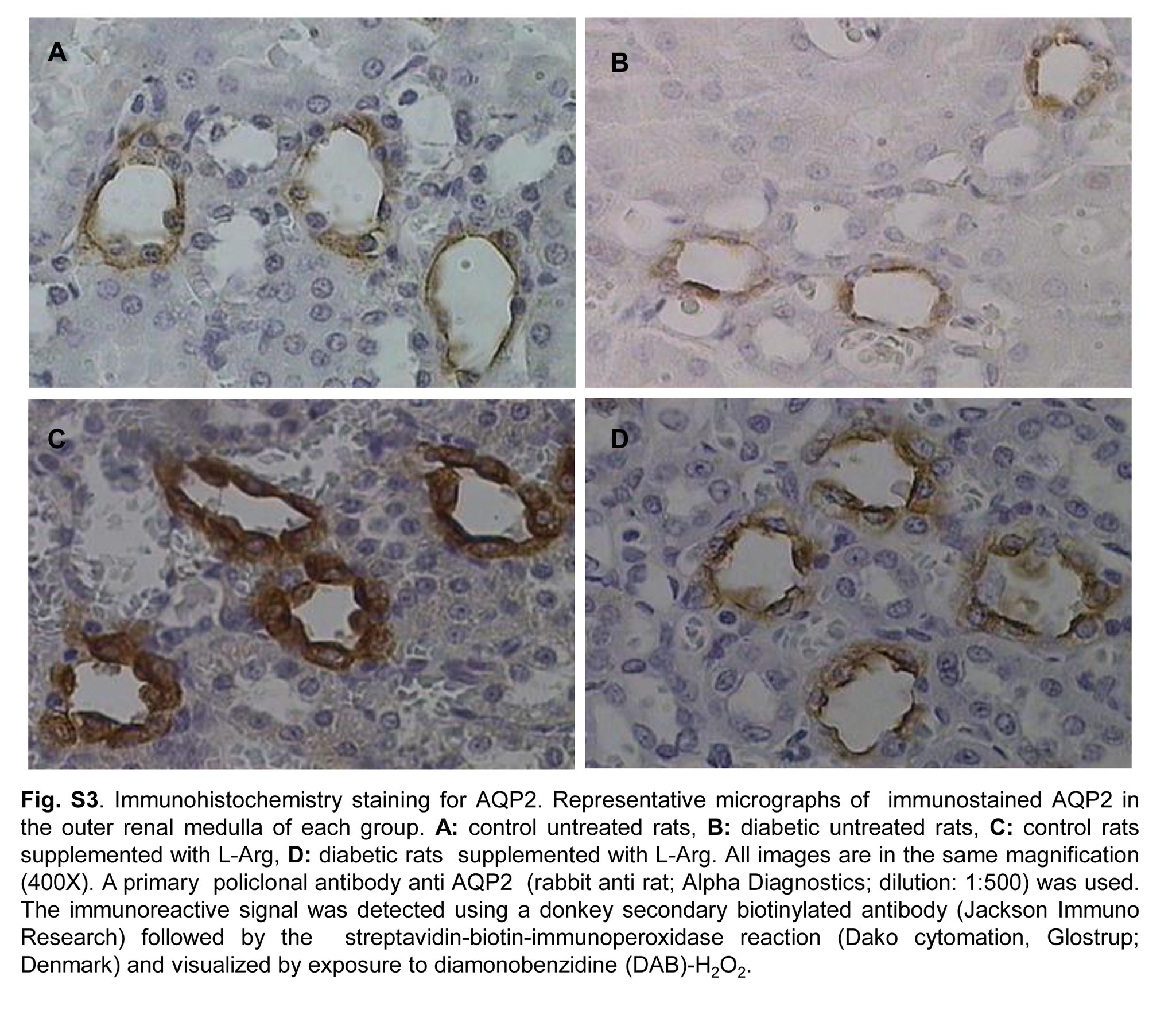

Supplement: Figure S3 — (TIF) [file pone.0104923.s003.tif]

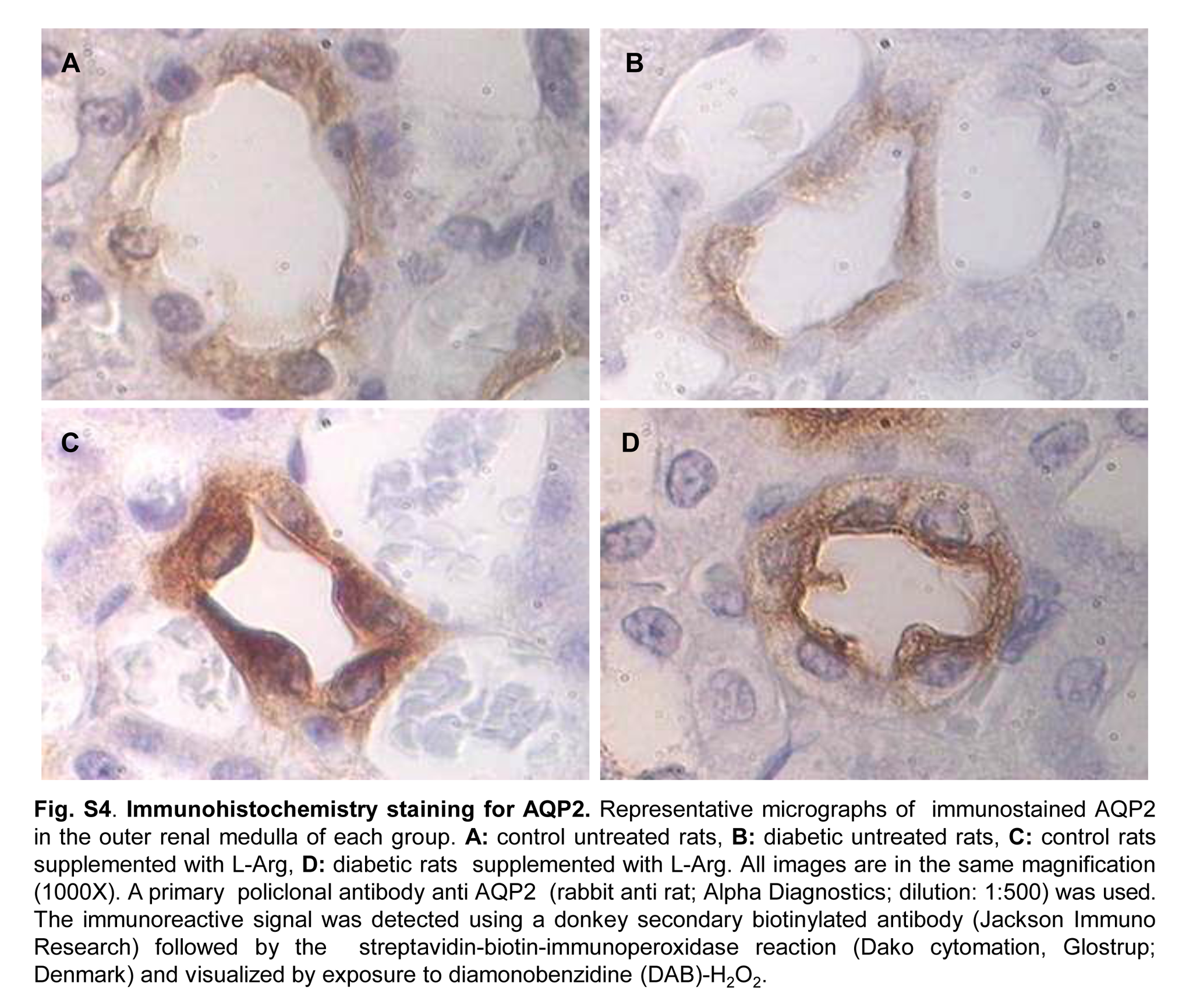

Supplement: Figure S4 — (TIF) [file pone.0104923.s004.tif]
